# Integrative multi-omics analysis and experimental validation identify molecular subtypes, prognostic signature, and CA9 as a therapeutic target in oral squamous cell carcinoma

**DOI:** 10.3389/fcell.2025.1629683

**Published:** 2025-07-09

**Authors:** Yun Zhao, Jing Yang, Yamei Jiang, Jingbiao Wu

**Affiliations:** ^1^ Department of Stomatology, Affiliated Hospital of North Sichuan Medical College, Nanchong, China; ^2^ Department of Stomatology, North Sichuan Medical College, Nanchong, China; ^3^ Demonstration Center for Experimental Teaching in Biomedicine, Chengdu Medical College, Chengdu, China

**Keywords:** oral squamous cell carcinoma, multi-omics analysis, machine learning, immunotherapy, prognosis, CA9

## Abstract

**Background:**

Oral squamous cell carcinoma (OSCC) is a challenging malignancy with poor prognosis despite therapeutic advancements. This study seeks to derive a precise molecular subtyping and prognostic model for personalized treatment strategies.

**Methods:**

Multi-omics data from TCGA cohort was analyzed using consensus clustering algorithms for subtype classification. Based on the classification, a multi-omics cancer subtyping signature (MSCC) model was constructed using machine learning methods. The model’s clinical utility was assessed by evaluating immune features and immunotherapy response. Potential therapeutic agents were identified through drug sensitivity analysis.

**Results:**

Three distinct OSCC subtypes with unique genetic and immunological profiles were identified. The MSCC model, developed using the StepCox [both]+plsRcox algorithm, demonstrated superior prognostic performance compared to existing models. High MSCC scores correlated with poor prognosis, reduced immune cell infiltration, and decreased likelihood of benefiting from immune checkpoint inhibitor therapy. Docetaxel and paclitaxel emerged as potential therapeutic candidates. *In vitro* experiments validated CA9 as a promising therapeutic target, with its knockdown significantly inhibiting OSCC cell proliferation and migration.

**Conclusion:**

This multi-omics analysis unveiled subtype-specific differences in OSCC and established an MSCC model for predicting prognosis and treatment response. These findings provide a foundation for early diagnosis, molecular subtyping, and personalized treatment strategies in OSCC.

## Graphical Abstract

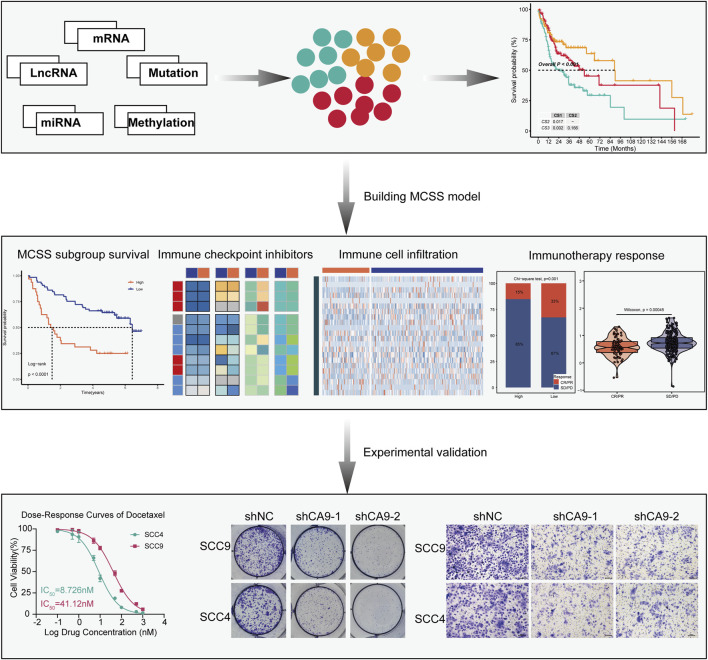

## 1 Introduction

Oral Cancer (OC), a frequent malignancy within the head and neck region, typically affects sites including the inner lip, dorsal tongue, gingiva, hard and soft palate, buccal mucosa, and mouth floor (Sarode et al., 2020). It rank among the most widely diagnosed cancer globally, with particularly high incidence rates in Asian nations where cases are increasing rapidly (Karunakaran and Muniyan, 2020; Nagao and Warnakulasuriya, 2020). The vast majority, around 90%, of OC cases are classified as Oral Squamous Cell Carcinomas (OSCC) (Tan et al., 2023). OSCC’s etiology is complex, evolving from normal cellular states through a sequence of pathological transitions to precancerous and cancerous states (Khan M. A. et al., 2023). Studies indicate that OSCC is subject to both genetic mutations and environmental exposures, resulting in the altered expression of proto-oncogenes and tumor suppressors (Irimie et al., 2018). Molecular mechanisms driving OSCC include somatic mutations, regulatory disruptions, epigenetic changes, and genomic variations (D'Souza and Saranath, 2017). DNA methylation is a key epigenetic process (Nasir et al., 2020), with dysregulated patterns potentially silencing tumor suppressor genes and accelerating tumorigenesis (Chamoli et al., 2021). mRNA modification imbalances have also been linked to the proliferation, migration, and invasiveness of OSCC cells (Liu et al., 2023). Moreover, mutations in specific genomic regions, such as the TERT promoter (Boscolo-Rizzo et al., 2023), and the p53 gene (Singh et al., 2022) are associated with increased OSCC aggressiveness. Comprehensive omics analyses of these alterations offer valuable perspectives on the molecular foundations of OSCC (Chai et al., 2020; Madhura et al., 2020), presenting novel avenues for its diagnosis and therapeutic intervention.

Preventing and detecting OSCC at an early stage can markedly enhance patient survival rates. Nonetheless, it is clear that the majority of OSCC cases are identified at later stages, resulting in a relatively poor 5-year survival rate (Abati et al., 2020). The clinical presentation of OSCC significantly impairs patients’ quality of life, affecting oral functionality, physical appearance, and mental health (Valdez and Brennan, 2018). Presently, the primary therapeutic approaches for OSCC encompass surgical excision, definitive radiotherapy, chemotherapy, or a combination of these modalities, contingent upon disease severity and individual patient conditions (Kim and Li, 2019; Pan and Rizvi, 2022). Despite these treatments, recurrence occurs in over half of OSCC patients, and among the diverse treatment strategies, immunotherapy has shown distinct benefits (Shetty et al., 2021). OSCC sidesteps immune surveillance by causing DNA damage, leveraging immune checkpoint facilitators, and emitting immunosuppressive cytokines (Tan et al., 2023). This presents an opportunity for the application of immunotherapy. In 2016, the FDA granted marketing approval for the inaugural class of immune checkpoint inhibitors (ICIs) targeting PD-1, marking a pivotal advancement in treating recurrent head and neck squamous cell carcinoma (HNSCC) (Cohen et al., 2019). These ICIs are an emerging principal therapeutic strategy in oncology, targeting the interactions of CTLA-4 and PD-1 along with its ligand 1 (PD-L1) to achieve checkpoint blockade (Naimi et al., 2022). However, the heterogeneity of OSCC and variability in ICI responsiveness among individuals continue to pose significant challenges in targeted therapies for OSCC patients, underscoring the pressing need to discover novel therapeutic targets. Precision medicine, integrating OMICS data with machine learning (ML), has revolutionized personalized treatment for OSCC, offering sophisticated molecular classifications and predictive models (Malcangi et al., 2023; Sultan et al., 2020). Our study integrated multi-omics data from patients with OSCC, including expression profiles of mRNA, lncRNA, miRNA, genomic mutations, and epigenetic DNA methylation. We employed 10 multi-omics integration strategies to establish a comprehensive consensus molecular subtyping of OSCC. Based on the characteristic genes identified among different subtypes, we constructed a classification model named multi-omics cancer subtyping signature (MSCC) using 10 ML algorithms. We evaluated the prognostic prediction performance of the MSCC model in training and independent validation datasets, and the results demonstrated that MSCC had significant prognostic value. Furthermore, MSCC exhibited strong performance in predicting responses to immunotherapy and drug therapy. Collectively, the OSCC molecular subtypes and MSCC classification model constructed in this study offer important novel insights and references for precise stratification and personalized treatment of this malignancy. We believe that these research findings will contribute to the optimization and innovation of future OSCC diagnosis and treatment strategies, ultimately benefiting more patients.

## 2 Data and methodology

### 2.1 Data pre-processing

We initially downloaded an integrated multi-omics dataset of OSCC from the TCGA-HNSC dataset, including transcriptome, methylome, somatic mutation, and clinical profiles. 316 OSCC clinical samples were included in the dataset. After excluding samples with survival time ≤0 days and duplicate entries, 314 valid clinical samples were retained. Among these, mRNA expression data were available for 305 cases, DNA methylation data for 310 cases, and somatic mutation data for 299 cases. By calculating the complete intersection of all four omics datasets (clinical, mRNA, methylation, mutation), 294 samples were ultimately obtained. The cohort included patients with a mean age of 61.2 years, with 45% T1-T2/55% T3-T4 stages, 60% lymph node metastasis, mostly moderately/poorly differentiated squamous cell carcinoma, a median follow-up of 626.5 days, and 46.3% mortality. Comparative analysis of clinical characteristics between the 294 included samples and 20 excluded samples demonstrated that the exclusion process did not introduce significant selection bias (mean ages: 61.2 years vs 58.2 years; mortality rates: 46.3% vs. 45.0%; median survival times: 626.5 days vs. 644.0 days, with all differences being statistically nonsignificant). We utilized the TCGABiolinks software package to obtain the transcriptional profiles of mRNA and lncRNA, and with miRNA annotations refined through the miRBaseVersions.db package. Somatic mutations data were extracted via TCGABiolinks and analyzed using the maftools package.

Additionally, we incorporated OSCC information from two other sources: the GEO datasets GSE65858 and GSE41613. For the GSE65858 cohort, we specifically selected patients with OSCC of the head and neck, excluding cases involving non-oral sites such as the hypopharynx, larynx, lips, tonsils, and oropharynx. Ultimately, 83 patients with pure oral squamous cell carcinoma were included, ensuring the homogeneity of the cohort. The GSE41613 cohort included 97 patients with oral squamous cell carcinoma. After downloading the these datasets from the official website, the limma package was used to process the data for background correction, log2 transformation, and quantile normalization. In cases where multiple probes mapped to a single gene symbol, the probe with the highest expression level was retained for gene expression annotation. And the GSE41613 and GSE65858 were merged into the META cohort, and batch effects were removed using the sva package for subsequent validation analysis.

### 2.2 Multiomics consensus ensemble analysis

Data integration was effectively achieved by matching samples ID (n = 294) from TGCA cohort including 5 dimensions. Preprocessing included log2 transformation of TPM values and CpG island probe selection for methylation data, and mutations were defined by non-synonymous categories: frameshift, insertion or deletion, in-frame insertion or deletion, nonsense or missense or nonstop mutation, or splice site or translation start site mutation.

Multi-Omics Integration and Visualisation for Cancer Subtyping (MOVICS) integrates 10 advanced multi-omics clustering algorithms (included SNF, CIMLR, PINSPlus, NEMO, COCA, moCluster, LRAcluster, ConsensusClustering, IntNMF, iClusterBayes), enabling the characterisation and comparison of identified subtypes from multiple perspectives, such as somatic mutations and genomic alterations, to achieve the most commonly used downstream analyses in cancer subtyping (Lu et al., 2021). Gene features selection was performed by the “getElites” function of MOVICS. For mRNA, lncRNA, miRNA, and DNA methylation, the “getElites” function with the “mad” parameter was used to select the top 2,000 most variable genes. For mutation data, “oncoPrint” in the maftools package prioritized top 1,000 mutated genes, followed by the “getElites” function with the “freq” parameter and an “elite.pct = 0.15” threshold to filter genes with mutation frequencies in the 15th percentile. The selected genes were then combined with clinical data for Cox proportional hazards regression analysis (using a p < 0.01 threshold) to enable subsequent prognostic stratification.

Further, the “getCluster2” function in MOVICS was used to define the optimal clustering number for OSCC subtypes classification by cluster prediction indices (CPI), gaps statistics, and silhouette score, in conjunction with information from previous studies on OC. Subsequently, the “getMOIC” function was applied for cluster analysis, which includes 10 clustering algorithms. Clustering results were merged using the consensus clustering concept with the “getconsensus susMOIC” function to enhance the robustness. Subsequent integration utilized hierarchical clustering parameters (“distance” = “euclidean”, and “linkage” = “average”) to produce definitive cluster partitions.

### 2.3 Analysis of features and stability of consensus subtypes

Gene set variation analysis (GSVA) was utilized to enrich characteristics associated with subtype Hallmark, KEGG pathways, and features related to immunotherapy and radiotherapy. These gene characteristics are derived from Explore the Molecular Signatures Database (MSigDB). Subgroup-specific immune checkpoints (ICs) distribution was evaluated, with tumor immune and stromal score of the tumor tissue calculated via ESTIMATE R package. The DNA methylation score of tumor-infiltrating lymphocytes was calculated, and immune microenvironment cell enrichments were profiled using GSVA. The construction of regulons was accomplished through the analysis of the Reconstruction of Transcriptional regulatory Networks and analysis of regulons (RTN) R package, which includes the collection of 23 inducible/repressible target-associated transcription factors (TFs) and 71 candidate regulators related to cancerous chromatin remodeling. Cluster robustness was verified through TCGA validation cohort analysis using subtype-specific molecular markers. A list of 150 genes consistently upregulated across subtypes was selected as the feature gene list, and the stability of each subtype was verified in the TCGA cohort using the nearest template prediction (NTP) algorithm. Additionally, the NTP algorithm and the partition around medoids (PAM) algorithm were employed to classify cell lines corresponding to different subtypes, and the kappa statistic was used to assess consistency.

### 2.4 Establishment of multiomics cancer subtyping signature

To promote comparability across cohorts, all data were preprocessed with Z-scoring. Subsequently, to evaluate the relationship between MCSS, immunotherapy, and prognosis, the TCGA cohort with comparably complete treatment information was selected as the training set, while the GSE41613 and GSE65858 cohorts served as validation sets. In the TCGA cohort, univariate Cox regression (uniCox) analysis was performed on differentially expressed genes across different OSCC subtypes. Genes with P < 0.05 and consistent hazard ratios across all cohorts were identified as candidate genes. Furthermore, an MCSS with high precision and generalizability was constructed by integrated 10 ML algorithms: StepCox, plsRcox, RSF, SuperPC, Ridge, Enet, CoxBoost, Lasso, GBM, and survivalSVM. The TCGA cohort functioned as the initial training set during the model construction phase. Initial features were selected using the stepwise Cox, CoxBoost, Lasso, and Enet algorithms, followed by the construction of an MCSS model with the best concordance index (C-index) from the 101 models combined from the aforementioned 10 algorithms. For the stepCox algorithm, all possible directional parameter combinations, including “both, “backward,” and “forward,” were calculated. For the Enet algorithm, alpha was varied from 0.1 to 0.9. The genes identified after MCSS screening are considered as signature genes for the model. If the number of selected genes is less than three, the model is excluded. The MCSS model is further validated in the validation cohort. The average C-index for each model is calculated, and the model with the highest value is considered the optimal model for constructing the MCSS model. Finally, multivariate Cox regression analysis is performed to generate MCSS-associated scores for each patient.

### 2.5 Prognostic value and clinical application analysis of MCSS

The generated MCSS model underwent multivariate Cox analysis on samples in the GSE41613, GSE65858, TCGA, and META cohorts. The MCSS score threshold was defined using the “surv-cutpoint” function, and samples were partitioned into high/low MCSS groups. The value of MCSS in predicting prognosis was estimated using K-M survival curves. Additionally, 20 OSCC-related prognostic features from research were systematically collected within the past 2 years and evaluated their risk scores against the C-index of MCSS. Finally, MCSS, Stage, Tstage, Nstage, Mstage, and Gender were assessed using multiple variables.

### 2.6 Correlation analysis of MCSS immunomodulators in multi - Omics

From a selection of 78 immunomodulatory genes (IMs), available genes were filtered based on criteria referenced from Thorsson et al. (2018), resulting in 67 genes and 9,058 samples. The median expression level of IMs in MCSS samples was calculated and normalized. The Bioconductor package IlluminaHumanMethylation450kanno.ilmn12.hg19 and IlluminaHumanMethylation27kanno.ilmn12.hg19 were used to map DNA methylation probes to genes. Within each MCSS group, Spearman correlation between gene expression and its corresponding probes was determined, retaining probe sets with consistent correlation signs for improved accuracy. Probe clusters were filtered to ensure that probes were uniquely assigned to a single cluster, were within a 10 kb size and had uniform correlation signs. The final correlation value for each cluster was derived by averaging individual probe correlations, and if multiple clusters linked to the same gene were further averaged. Copy number alterations, including amplifications and deletions, were analyzed across 8,461 tumors using PanCan GISTIC2.0 with ISAR-corrected Affymetrix SNP6.0 array data. The proportions of each variation type were compared across MCSS groups, and then the difference between the frequency of amplifications/deletions and expected frequency (total frequency of amplifications/deletions in all tumour samples) were calculated in each IMs.

### 2.7 MCSS immune infiltration analysis

Seven immune infiltration methods including CIBERSORT, MCPcounter, xCell, EPIC, estimate, TIMER, and quanTIseq were employed to evaluate the differences in immune cell types, and relative abundance.

### 2.8 Analysis of the prognostic value of MCSS for anti-PD-L1 therapy

We further utilized two cohorts receiving PD-L1 therapy (IMvigor210 and GSE78220) to explore the prognostic and predictive value of MCSS for PD-L1 treatment. The performance of MCSS in forecasting the prognosis of PD-L1 treatment was assayed by the time-dependent receiver operating characteristic (ROC) curve. The patients demonstrating complete/partial response (CR/PR) were classified as responders, while those with stable/progressive disease (SD/PD) were non-responders.

### 2.9 Screening and analysis of potential therapeutic drugs

The GSEA algorithm was implemented to profile the upregulated pathways in patients with high MCSS. Expression data for Human Cancer Cell Lines (CCL) were obtained from the Broad Institute’s CCL Encyclopedia (CCLE). Drug sensitivity data for CCL were acquired through CTRP v.2.0 and the PRISM repurposing dataset from the DepMap portal. Drug sensitivity was derived from predicted area under the ROC curve (AUC) value, which calculating by the calcPhenotype function from the R package “pRRophetic”. The Wilcoxon rank-sum test and Spearman correlation analysis were employed to determine the significant differences in sensitivity to six commonly used chemotherapeutic drugs for OSCC. A lower AUC value indicates greater sensitivity to drug therapy.

Expression and drug sensitivity data for 1,100 tumor cell lines were sourced from the DepMap database, including 47 OSCC cell lines. These data were analysed to investigate the relative expression levels of the target genes CA9 and SPINK6 in OSCC cells and utilized these expression levels to predict their sensitivity to the drug docetaxel. Additionally, we employed the Computational Estimation of Resistance and Sensitivity (CERES) method to predict the effect of target gene knockout on the proliferative capacity of OSCC cells. A lower CERES score indicates that the gene is more critical for cell survival and proliferation.

### 2.10 MTT assay

OSCC cell lines SCC9 (Cat No. B26673) and SCC4 (Cat No. B26674), from Sichuan Bio Biotech Co., Ltd. (http://htycbio.com/IVDyuanliao/), cultured in DMEM/F12 + 10% FBS (37°C, 5% CO_2_). Docetaxel (Cat No. HY-B0011; purity ≥99.94%) and Pembrolizumab (Cat No. HY-P9902; purity ≥99.17%) were purchased from MedChemExpress (https://www.medchemexpress.cn). Docetaxel was dissolved in dimethyl sulfoxide (DMSO) to prepare a 10 mM mother liquor and store at −20°C. Pembrolizumab was dissolved in phosphate buffered saline (PBS) to prepare a 10 mg/mL mother liquor, which was stored at 4°C.

SCC4 and SCC9 cells were seeded into 96-well plates at a density of 4,000 cells/well and cultured overnight for cell attachment. For Docetaxel treatment groups, a concentration gradient of 0.1–1,000 nM (0.1, 0.5, 1, 5, 10, 50, 100, 500, and 1,000 nM) was applied for 72 h. For Pembrolizumab treatment groups, a concentration gradient of 0.1–200 μg/mL (0.1, 0.5, 1, 5, 10, 25, 50, 100, and 200 μg/mL) was applied for 72 h. Control groups received equivalent volumes of solvent (DMSO for Docetaxel control with final concentration <0.1%; PBS for Pembrolizumab control). After drug treatment, 20 μL of MTT solution (5 mg/mL in PBS) was added to each well, followed by 4 h of incubation at 37°C. The culture medium was carefully removed, and 150 μL of DMSO was added to dissolve the formazan crystals. Absorbance was measured at 570 nm using a microplate reader. Cell viability percentages were calculated relative to the solvent control group (set as 100%).

### 2.11 Cell culture and CA9 knockdown

CA9 knockdown was achieved by transfecting cells with CA9-specific or control shRNA via Lipofectamine 3,000 (Invitrogen) as directed, with efficacy validated by Western blot 48 h post-transfection. The CA9-specific shRNA sequences were listed below:

shRNA-1: (Forward: 5′-CCGGCTACCTGAAGTTAAGCCTAAACTCGAGTTTAGGCTTAACTTCAGGTAGTTTTTG-3′, Reverse: 5′-AATTCAAAAACTACCTGAAGTTAAGCCTAAACTCGAGTTTAGGCTTAACTTCAGGTAG-3′)

shRNA-2: (Forward: 5′-CCGGCAGCCGCTACTTCCAATATGACTCGAGTCATATTGGAAGTAGCGGCTGTTTTTG-3′, Reverse: 5′-AATTCAAAAACAGCCGCTACTTCCAATATGACTCGAGTCATATTGGAAGTAGCGGCTG-3′).

### 2.12 Western blot analysis

Total protein was extracted from SCC9 and SCC4 cells using RIPA lysis buffer with protease inhibitors. After protein quantitation via BCA, 30 μg equivalent protein were resolved by 10% SDS-PAGE and transferred onto PVDF membranes. The membranes were blocked for 1 h at room temperature (RT) with 5% non-fat milk, and then incubated overnight at 4°C with primary anti-CA9 (1:1,000) and β-actin (1:5000) antibodies. After washing, the membranes were incubated with HRP-conjugated secondary antibodies for 1 h at RT. Protein bands were visualized using ECL and quantified by ImageJ. The CA9 antibody (Cat No. 11071-1-AP) were purchased from Proteintech (https://www.ptgcn.com/).

### 2.13 Colony formation assay

To assess the effect of CA9 knockdown on cell proliferation, colony formation assays were conducted. CA9-knockdown or control SCC9 and SCC4 cells were plated in 6-well plates (1,000 cells/well) and maintained for 14 days with medium renewal every 3 days. Colonies were fixed (4% paraformaldehyde, 15 min) and stained (0.1% crystal violet, 20 min), followed by microscopic quantification of colonies (>50 cells).

### 2.14 Transwell invasion assay

To evaluate the invasive ability of oral OSCC cells after CA9 gene knockdown, the Transwell invasion assay was performed. 50 μL of the Matrigel matrix gel (diluted 1:8 with serum-free medium) was added to the upper chamber of the Transwell insert, and then incubated at 37°C for 4 h. SCC9 and SCC4 cells in the logarithmic growth phase were resuspended in serum-free medium to a concentration of 2 × 10^5 cells/mL. Subsequently, 100 μL of the cell suspension (shNC, shCA9-1, and shCA9-2 groups) was seeded into the upper chamber, while 600 μL of complete medium containing 10% fetal bovine serum was added to the lower chamber as a chemoattractant. After incubation at 37°C with 5% CO_2_ for 24 h, the upper chamber was rinsed with PBS, and non-invaded cells on the upper membrane were removed with a cotton swab. The cells were then fixed with 4% paraformaldehyde for 30 min, stained with 0.1% crystal violet for 20 min, and rinsed with deionized water before air-drying. Invasive cells were observed under an optical microscope, and five random fields of view were photographed.

### 2.15 Wound healing assay

CA9-knockdown or control SCC9 and SCC4 cells were cultured in 6-well plates. Once the cells reached 90% confluence, we made a scratch on the cell monolayer using a 200 μL pipette tip. After that, we rinsed the wells with PBS to get rid of the cell debris. Then, we replaced the medium with serum-free medium. We captured images of the wound areas at the starting point (0 h) and 24 h later using an inverted microscope. Wound closure rates were calculated in ImageJ by comparing wound dimensions over time.

### 2.16 Statistical analysis

For data obtained from public databases, all analyses were performed in R.4.1.0. Unpaired Student’s t-tests were used to evaluate differences between two groups for normally distributed data, whereas the Wilcoxon rank-sum test was applied for non-normally distributed data. For comparisons involving more than two groups, parametric and nonparametric variables were tested by one-way ANOVA and KruskalWallis test, respectively. Differential expression analysis was analyzed by the limma package.

All experiments were conducted in triplicate, with data expressed as mean ± standard deviation (SD). Statistical analysis was performed using GraphPad Prism 8.0, employing Student's t-tests for group comparisons. Significance was defined as P < 0.05.

## 3 Results

### 3.1 Integration of multi-omics data of OSCC

**FIGURE 1:**
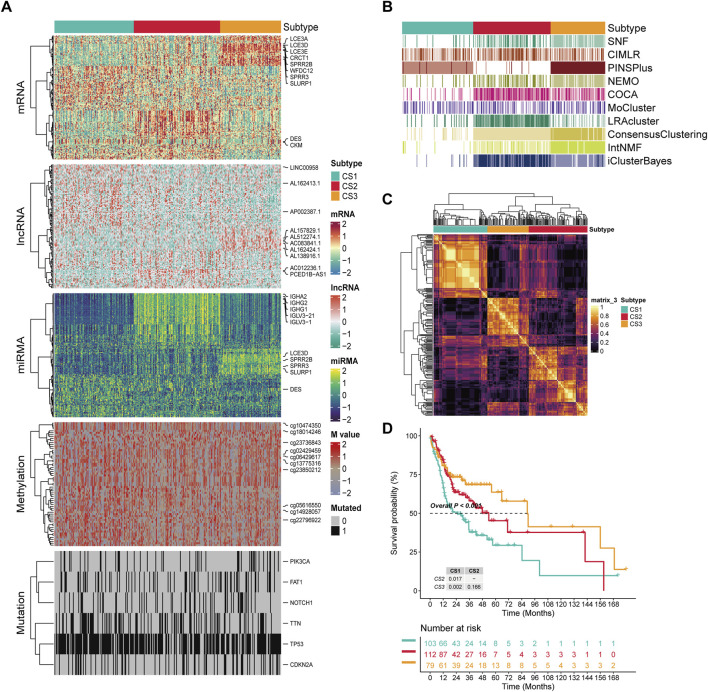
The multiomics integrative consensus subtypes of OSCC. **(A)** Comprehensive heatmap of consensus ensemble subtypes, including mRNA, lncRNA, miRNA, DNA CpG methylation site, and mutant gene. **(B)** Clustering of OSCC patients through 10 cutting-edge multiomics clustering methods. **(C)** Consensus clustering matrix for three novel prognostic subtypes based on the 10 algorithms. **(D)** Different survival outcomes among the three subtypes. Dashed line: median survival time.

We conducted a clustering analysis using information from five levels: mRNA, lncRNA, and miRNA expression, along with DNA methylation and mutation profiles. The optimal number of subgroups was determined through CPI, gap statistics, and silhouette scores, followed by ten multi-omics consensus clustering algorithms, which identified three prognostic OSCC subtypes (CS1, CS2, and CS3) (Figures 1A–C). The top ten overall survival (OS)-related factors from each dimension are displayed on the right. For example, six mutated genes with significantly differences across the three subtypes were identified, including PIK3CA, FAT1, NOTCH1, TTN, TP53, and CDKN2A. These subtypes exhibit different OS outcomes, indicating that the classification of OSCC subtypes has clinical significance (P < 0.001; Figure 1D). K-M analysis indicated that CS3 patients had the longest median survival (90 months), exceeding CS2 patients (54 months) by 36 months (P _CS3/CS2_ = 0.166). Both of these groups significantly outlive CS1 patients, who have a median survival of 28 months (P _CS3/CS1_ = 0.002, P _CS2/CS1_ = 0.017).

### 3.2 Characterization of OSCC integration consensus molecular subtypes

**FIGURE 2:**
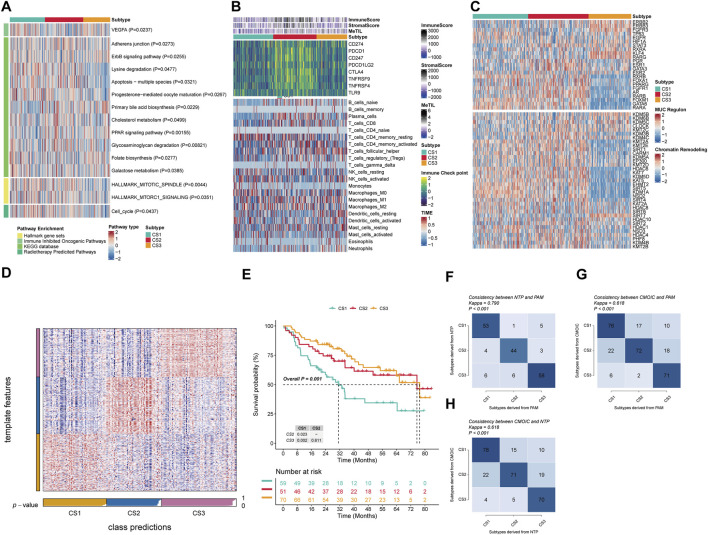
Molecular landscape and validation of OSCC CSs. **(A)** Pathway enrichment of the three isoforms for different treatment-associated features and oral cancer-associated features. **(B)** Regulator activity profiles of 23 TFs in the three subtypes (upper panel) and potential regulators associated with chromatin remodeling (lower panel). **(C)** Immunoprofiling in the TCGA cohort. The top annotation of the heatmap shows the immune enrichment score, stromal enrichment score, and DNA methylation of tumor-infiltrating lymphocytes. The top part of the graph shows the expression of typical immune checkpoint genes, and the bottom panel shows the enrichment levels of 22 TME-associated immune cells. **(D)** Validation of OSCC CS in recent templates of the META cohort. **(E)** Survival analysis of OSCC CSs in the META cohort. **(F)** Consistency analysis of CSs by PAM in the META cohort. **(G)** Consistency analysis of CSs by PAM in the TCGA cohort. **(H)** Consistency analysis of NTP in the TCGA cohort.

In this study, GSVA was applied to measure the enrichment scores of subtype-specific features in samples, characterizing the biological functions of OSCC subtypes classified based on molecular expression levels. Our findings revealed distinct molecular characteristics across different subtypes (Figure 2A). KEGG pathway analysis indicated significant alterations in metabolic and signaling pathways among the subgroups (P < 0.05). Notably, cholesterol synthesis and metabolism were likely suppressed in the CS1 subgroup, with Primary bile acid biosynthesis, Cholesterol metabolism, and PPAR signaling pathways all exhibiting significant downregulation. In contrast, the CS2 subgroup showed significant positive enrichment of Adherens junction, Cholesterol metabolism, and Glycosaminoglycan degradation pathways, suggesting activation of cell adhesion mechanisms, cholesterol metabolism, and glycosaminoglycan degradation. The CS3 subgroup, however, demonstrated a significant downregulation of Primary bile acid biosynthesis compared to the other subgroups, which showed an upregulation (P = 0.0229). Furthermore, we observed a gradual increase in negative enrichment scores for the ErbB signaling pathway, Lysine degradation, Apoptosis across multiple species, and Progesterone-mediated oocyte maturation as the disease progresses from CS1 to CS3. This suggests that the activity of pathways related to cell proliferation and death may be increasingly inhibited with disease progression. The differential signals related to cell proliferation showed significant differences in the Hallmark gene set, and as progression occurs from CS1 to CS3, we noted an increasingly enhanced inhibitory trend in mitotic spindle and mTORC1 signaling, potentially related to changes in cell cycle control during tumor development. The response to specific treatments also varied significantly between subtypes (P<0.05). CS1 significantly enriched pathways for immune suppression in cancer and radiotherapy, while CS2 showed insensitivity to immunotherapy, and CS3 showed insensitivity to cell cycle-based radiotherapy. Compared with CS1, CS2 significantly negatively enriched with the immune inhibited oncogenic pathways (VEGFA), whereas CS3 exhibited significant negative enrichment in the radiotherapy-predicted pathway (Cell cycle).

To more in-deep investigate transcriptomic differences, we analyzed 23 TFs and potential regulators related to cancer chromatin remodeling in OSCC (Figure 2B). Notably, we found significant activation of FOXM1, GATA6, and RARA in CS1, and ESR1, GATA3, FGFR1, and AR in CS2. Additionally, STAT3, KLF4, RARG, ESR2, and GATA3 were specifically enriched in CS1, and RXRB, ESR2, GATA3, FOXA1, PPARG, FGFR1, RARB, AR, FOXM1, RARA, and GATA6 were significantly negatively enriched in CS3. These regulators differences further highlight subtype-specific transcriptional regulatory mechanisms, suggesting that epigenetic alterations may play a pivotal role in molecular stratification. For example, CS3 showed a significant negative enrichment of HDAC6, KAT7, KDM5D, KDM5, EHMT2, SIRT5, KDM1A, and NSD2. Given the established role of immune functions in tumor development, we quantified the level of immune cell infiltration in the microenvironment. The results indicated significant changes in immune cells across subgroups; CS2 demonstrated a significant increase in immune cell infiltration compared to the other two subgroups, with immune checkpoint molecules PDCD1, CD247, PDCD1LG2, CTLA4, TNFRSF9, and TNFRSF4 also being highly expressed in CS2 (Figure 2C). This indicates that immunotherapy is a viable option for CS2 patients. Specifically, T cells CD4 memory resting, NK cells activated, and Monocytes showed significant increases in CS1, while in CS2 there was also a significant increase in B cells naive, T cells CD8, T cells CD4 memory activated, and Macrophages M1. Meanwhile, CS3 exhibited a notable decrease in Plasma cellsbut an increase in T cells follicular helper and Dendritic cells resting.

To verify the stability of the molecular subtypes determined by multi-omics analysis, we first applied the NTP algorithm. In the TCGA cohort, we observed that the prognostic predictions of the three subtypes were consistent with the molecular subtypes we determined, especially the CS3 subtype, which showed the most favourable prognosis of all subtypes (P = 0.001) (Figures 2D–E). At the same time, we also used the NTP classifier and PAM classifier for validation in the TCGA cohort, and assessed consistency using the kappa statistic. The kappa values from the mutual comparison of the NTP, PAM, and multi-omics subtypes (CMOIC) algorithms were all greater than 0.6 (P < 0.001) (Figures 2F-H). These results together provided strong evidence for the high consistency of the identified subtypes, providing strong support for their stability and reliability.

### 3.3 MCSS risk stratification system development and predictive value assessment

**FIGURE 3:**
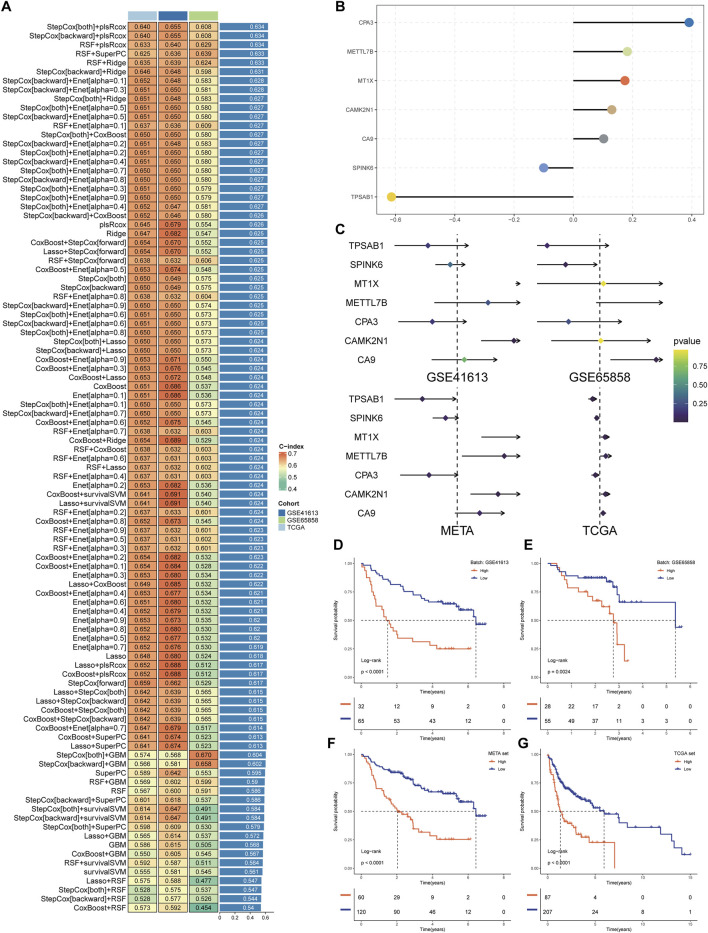
Establishment of the MCSS. **(A)** Heat map of 99 combined machine learning algorithms. Based on 101 algorithms of 10 combinations of machine learning methods, the C-index of each model was calculated by GSE41613, GSE65858, and TCGA cohort, and sorted by average C-index. **(B)** Multivariate Cox model coefficients of pivotal genes selected by StepCox [both]+plsRcox algorithm. **(C)** Results of univariate Cox regression analysis of pivotal genes in the training and validation cohort. **(D–G)** Survival analysis of OSCC patients with high and low MCSS in GSE41613, GSE65858, META and TCGA cohorts.

In the development and evaluation of the MCSS risk stratification system, we applied uniCox analysis to select 32 candidate genes with significant expression correlations to OS (P < 0.05) from TCGA, which were then incorporated into an integrated framework for executing MCSS. Within the TCGA training cohort, the model composed of StepCox [both] and plsRcox among 101 algorithm combinations maintained the highest average C-index (0.640), indicating its more accurate predictive performance (Figure 3A). The most valuable prognostic gene was identified by the StepCox algorithm, and the most valuable model was selected by the plsRcox algorithm, ultimately constructing an MCSS model with 7 model genes (Figures 3B,C). The uniCox analysis results of the 7 genes across multiple datasets are shown in Figure 3C. Analysis of MCSS scores indicated that patients with higher MCSS scores had significantly poorer prognosis in the TCGA, GSE41613, GSE65828, and META cohorts (Figures 3D–G).

**FIGURE 4:**
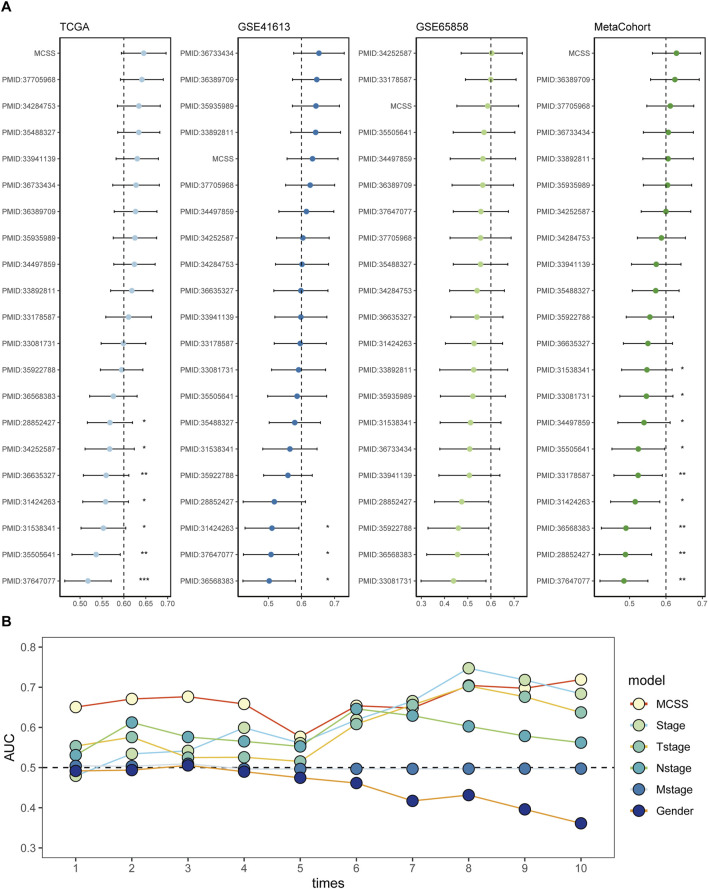
Assessment of the predictive value of the MCSS model for prognostic purposes. **(A)** Comparison of MCSS features among the MCSS model and 21 other published models in TCGA, GSE41613, GSE65858, and META cohort. **(B)** Cox analysis of the MCSS model combined with MCSS, Stage, Tstage, Nstage, Mstage, and Gender features.

To more accurately and comprehensively evaluate the reliability of the MCSS model, we systematically reviewed literature on OSCC prognostic models published in the past 5 years. Eventually, 20 features were included, systematically representing diverse physiological pathways, including ferroptosis, oxidative stress, immune therapy response, immune infiltration, and glycolysis. Feature comparison analysis across four datasets found that the MCSS model consistently showed high consistency in the C-Index (C-index >0.6) (Figure 4A). Furthermore, survival analysis results also indicated that MCSS had a higher AUC value (Figure 4B). This finding confirms the applicability of the MCSS model as a powerful prognostic tool. Notably, even after considering advanced features based on Stage and Tstage, the MCSS model still maintained a good AUC value, emphasizing its robustness and practicality in cancer prognosis assessment. These results not only validate the effectiveness of the MCSS model but also demonstrate its superiority and clinical practicality in cancer prognosis assessment through robust performance across multiple independent datasets.

### 3.4 Immune landscape of MCSS

**FIGURE 5:**
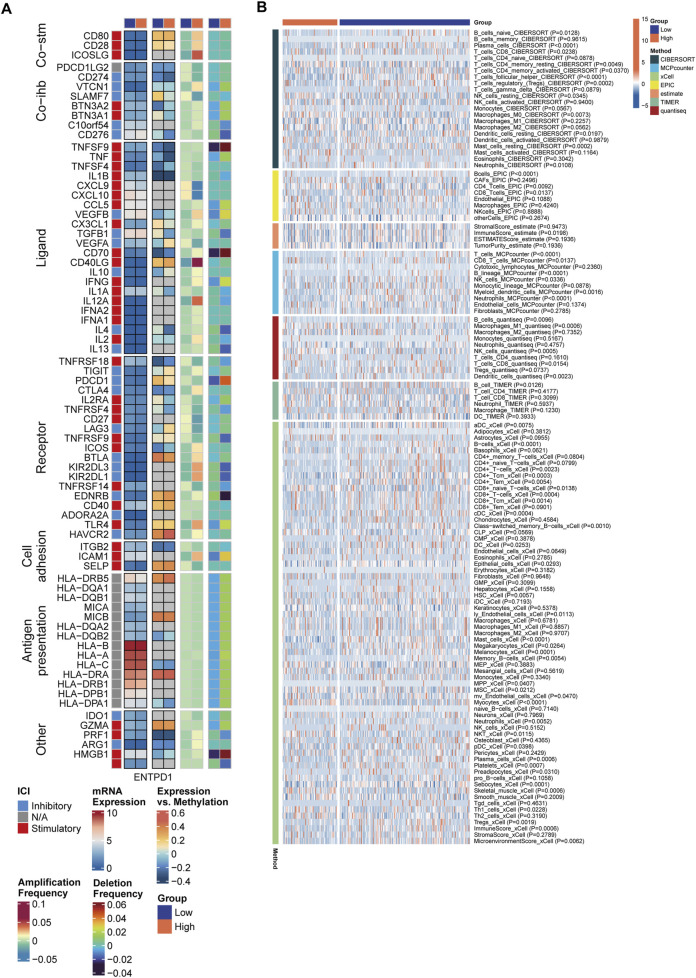
Differences in immune characteristics between MCSS subgroups. **(A)** Correlation between MCSS and immunomodulators. From left to right: mRNA expression (median normalized expression levels), expression versus methylation (gene expression correlation with DNA methylation beta-value), amplification frequency, and deletion frequency for regulators. **(B)** Heatmap showing the relative abundance differences of immune cell types.

In the exploration of the immunological landscape of the MCSS, IMs are recognized for their potential to alter host immune regulatory responses by either promotion or inhibition, affecting various targets and marking a future direction in cancer therapy (Wu et al., 2022). We investigated the expression and regulatory mechanisms of IMs in the tumor immune microenvironment (TIME) through epigenetic and miRNA mechanisms. Among the 65 key immunomodulatory genes, 22 genes (33.8%) exhibited significant dysregulated expression patterns between the low MCSS and high MCSS groups, suggesting a close association between MCSS scores and the remodeling of the TIME (Figure 5A). Compared to the low MCSS group, the high MCSS cohort exhibited marked upregulation of IMs expression including CD276, VEGFB, TGFB1, CD70, and MIC (P < 0.05); and SLAMF7 and CD28 showed significant downregulation (P < 0.05) (Supplementary Table S1). Concurrently, immune receptors such as TIGIT, CD27, TNFRSF18, TNFRSF4, ICOS, and BTLA were significantly downregulated, alongside a significant reduction in the expression of cytokines such as CD40LG, IFNG, and IL2 (P < 0.05). However, a greater number of IMs in high MCSS groups and low MCSS group are negatively correlated with DNA methylation, such as PDCD1LG2, CD274, BTN3A2, TNFSF9, TNF, TNFSF4, IL1B, CD70, TNFRSF18, and PRF1, indicating that gene silencing and immune system activation may be suppressed. We also observed that gene expression and DNA methylation of SLAMF7, TGFB1, VEGFA, and LAG3 showed opposite correlations between the high and low MCSS group, which may be associated with the development of OSCC. Additionally, comparative analysis between groups revealed that CNV-driven genomic alterations were significantly enriched in TNFSF9, CD70, CD40LG, and TLR4 (P < 0.05).

We further assessed the levels of immune cells in the two MCSS groups using 7 immune infiltration algorithms. The heatmap revealed significant differences in the composition of immune cells between subgroups (Figure 5B). A more detailed examination of immune cell abundance found that patients in high MCSS group, had significantly reduced levels of various immune cell subsets compared to the low MCSS group, including Neutrophils, Plasma cells, Platelets, Mast cells, CD8^+^ Tcm, CD8^+^ T cells, CD8 T cells, CD4^+^Tem, CD4^+^Tcm, CD4^+^ T cells, T cells, B cells, B lineage, T cells regulatory (Tregs), and T cells CD4 memory activated (P < 0.05) (Supplementary Figure S1). Additionally, the immune score of the high MCSS group was significantly reduced (P < 0.05). In contrast, NKT, NK cells resting, NK cells, CD4 Tcells, Macrophages M1, and T cells CD4 memory resting exhibited higher expression in high MCSS group (P < 0.05). This shows that the high MCSS group may have an immunosuppressive microenvironment, thereby increasing the risk of OSCC.

### 3.5 Prediction of immunotherapy response in MCSS

**FIGURE 6:**
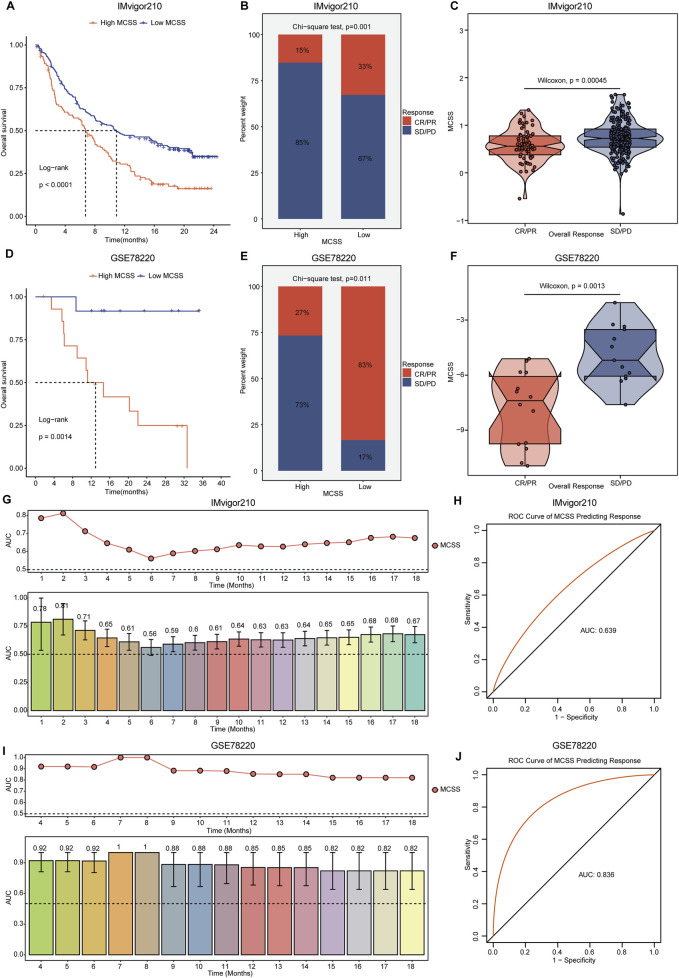
Assessment of immunotherapy response by MCSS model in OC patients. **(A–C)** Differences in anti-PD-L1 efficacy of MCSS model and immunotherapy response predicted by TIDE algorithm in IMvigor210 cohort. **(D–F)** Differences in anti-PD-L1 efficacy of MCSS models and immunotherapy response response predicted by the TIDE algorithm in the GSE78220 cohort. From left to right, the figure sequentially illustrates the OS differences, disparities in immunotherapy response, and variations in MCSS scores across groups with distinct immunotherapy responses in each cohort. They were compared by Wilcoxon test. *P<0.01, **P<0.01, ***P<0.001. **(G)** Prognostic time curves of the IMvigor210 cohort. **(H)** Immune response response accuracy of the IMvigor210 cohort. **(I)** Prognostic time curve of the GSE78220 cohort. **(J)** Immune response response accuracy of the GSE78220 cohort.

In the context of cancer treatment, a reduction in immune cells may suggest the need for new therapeutic strategies. Tumor immunotherapy, especially ICIs, has demonstrated remarkable efficacy in cancer treatment. Currently, anti-PD-L1 antibodies have shown effectiveness against various cancer types (Rasihashemi et al., 2022). Patients receiving anti-PD-L1 immunotherapy were selected to further validate the potential capabilities of the MCSS model. In the IMvigor210 and GSE78220 cohorts receiving immunotherapy, we observed that patients with high MCSS had poorer prognosis (Figures 6A,D), while those with low MCSS exhibited more favorable responses to immunotherapy (Figures 6B,C,E,F). To further assess the prognostic value of MCSS in forecasting the efficacy of anti-PD-L1 treatment, we employed time-dependent AUC curves for analysis. In the IMvigor210 cohort, MCSS achieved the highest AUC value (0.81) at 2 months of treatment, indicating its good performance in predicting prognosis (Figure 6G) and immune response (AUC = 0.639, Figure 6H). In the GSE78220 cohort, MCSS demonstrated excellent prognostic prediction ability, with AUC values reaching 1 at 7 and 8 months of treatment (Figure 6I). Moreover, the ROC curves for predicting response also confirmed the outstanding performance of the MCSS model in forecasting immunotherapy response (Figure 6J). Collectively, these results suggest that MCSS possesses remarkable capability in predicting both prognosis and immunotherapy response in patients receiving immunotherapy, highlighting its potential as a valuable predictive tool.

### 3.6 Potential therapeutic drug development for patients with high MCSS

**FIGURE 7:**
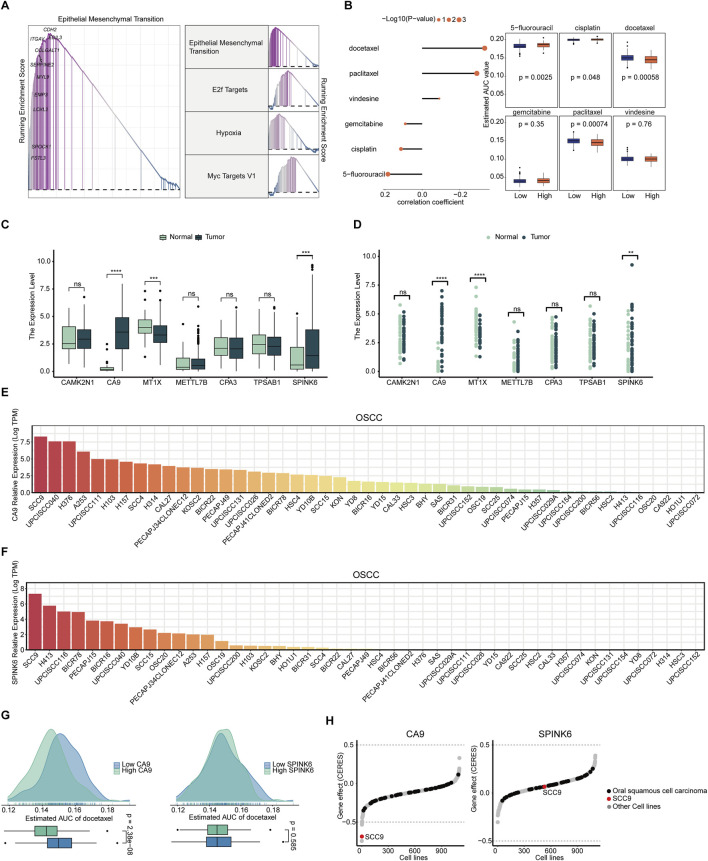
Screening and analysis of potential drugs in patients with high MCSS **(A)** Pathways significantly upregulated in the high MCSS group. **(B)** Correlation and differential analysis of drug sensitivity of potential drugs screened from CTRP and PRISM datasets. **(C,D)** Unpaired and paired differential expression analysis of potential target genes of drugs in normal and tumor tissues. *P < 0.05, **P < 0.01, ***P < 0.001, ****P < 0.0001. **(E)** Expression of CA9 gene in OSCC cell lines. **(F)** SPINK6 gene expression in OSCC cell lines. Data from CCLE; DepMap Public 22Q4. dataset. **(G)** Predicted effects of docetaxel on CA9 and SPINK6. **(H)** Gene effect (CERES) scores of CA9, SPINK6 in oral cancer cell lines. Data from CRISPR (AVANA) DepMap v22Q4 dataset.

In response to the high MCSS group’s poor response to immunotherapy, we further employed GSEA to analyze the signaling pathways related to its expression. Pathway enrichment analysis revealed significant upregulation in the high MCSS group, with the top four pathways having the highest enrichment scores being Epithelial Mesenchymal Transition, E2f Targets, Hypoxia, and Myc targets V1 (Figure 7A). Based on this, we used the CTRP and the PRISM approach to screen for possible curative drugs for high MCSS patients, and we conducted drug response analysis and differential evaluation for the two MCSS subgroups to identify drugs sensitive to the high MCSS group. We then performed Spearman correlation analysis between the AUC values and the MCSS risk scores. Ultimately, three potential drugs were identified with AUC values negatively correlated with the MCSS scores: docetaxel, paclitaxel, and vindesine. Docetaxel and paclitaxel both showed lower Estimated AUC values in the high MCSS group (P < 0.05) (Figure 7B). This indicates that these drugs have higher sensitivity to the high MCSS group, especially docetaxel, which has significant therapeutic potential.

The expression of candidate drug target genes in tissue of OSCC tumor and normal tissue was assessed. Unpaired and paired differential expression analysis results showed that CA9 and SPINK6 have higher expression in tumor tissue (P < 0.05) (Figures 7C,D), indicating their involvement in development of OSCC. DepMap analysis further confirmed the above results. CA9 and SPINK6 are abnormally activated in many OSCC cell lines, especially in SCC9, where the expression level exceeds 7.5 logTPM (Figures 7E,F). Therefore, we further analyzed the relationship between the most promising docetaxel and highly expressed genes in tumor cells. Docetaxel treatment showed a lower Estimated AUC in the high CA9 group (P < 0.05), but no effect in the high and low SPINK6 groups (P > 0.05) (Figure 7G). This indicates that docetaxel effectively inhibits CA9. We used the CRISPR loss-of-function screen from the DepMap database for analysis, focusing on the SCC9 cell line where both genes are highly expressed. According to the definition of CERES, each gene shows different tendencies as a potential oncogenes or tumor suppressor genes; the lower the CERES score of the gene effect, the lower the cell survival rate. Notably, CA9 has a negative gene effect in many OSCC cell lines, with the CERES score in SCC9 cells being lower than −0.5, indicating that SCC9 cells are highly dependent on the expression of CA9 (Figure 7H). On the other hand, SPINK6 tends to have a positive gene effect, indicating that it is not essential for the survival of OSCC cells. Overall, CA9 loss generally promotes OSCC cell death, and docetaxel’s inhibition of CA9 activation suggests promise for targeting OSCC cell survival and progression.

### 3.7 Validation of the immune therapy prediction ability of MCSS model and the therapeutic potential targeting CA9

**FIGURE 8:**
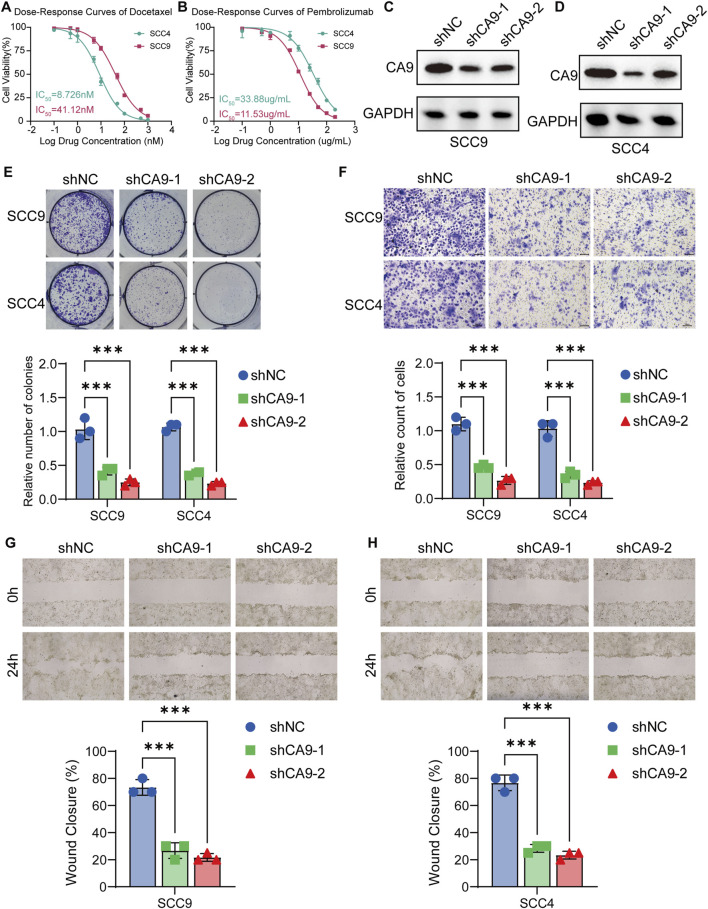
Validation of CA9 as a potential therapeutic target in OSCC cell lines **(A)** Analysis of drug sensitivity of OSCC to Docetaxel. **(B)** Analysis of drug sensitivity of OSCC to Pembrolizumab. **(C,D)** Western blot analysis of CA9 protein expression in SCC9 and SCC4 cells transfected with control shRNA or CA9-specific shRNA. GAPDH was used as a loading control. **(E)** Colony formation assay results for SCC9 and SCC4 cells transfected with control shRNA or CA9-specific shRNA. **(F)** Transwell invasion assay results of SCC9 and SCC4 cells transfected with control shRNA or CA9 specific shRNA. **(G,H)** Wound healing assay results for SCC9 and SCC4 cells transfected with control shRNA or CA9-specific shRNA. Data are presented as mean ± SD from three independent experiments. *P < 0.05, **P < 0.01, ***P < 0.001 (Student’s t-test).

To validate CA9’s role in OSCC progression and its therapeutic potential, we performed *in vitro* experiments on SCC4 (MCSS score = 2.686) and SCC9 (MCSS score = 2.200) with different MCSS scoring characteristics. The MCSS score was calculated based on the transcriptome expression data of OSCC cell lines in the DepMap database, using the seven characteristic genes and their corresponding coefficients of the MCSS model (Supplementary Table S2; Supplementary Figure S2). The results of drug sensitivity showed that the IC50 of SCC4 cells to docetaxel was 8.726 nM, significantly lower than the 41.12 nM of SCC9 cells, indicating that SCC4 cells have higher sensitivity to docetaxel (Figure 8A). This direct IC50 measurement result is highly consistent with our prediction based on AUC values, effectively verifying the accuracy of the MCSS model in predicting chemotherapy sensitivity in OSCC. In addition, we also tested the sensitivity of these 2 cell lines to the ICIs pembrolizumab. The IC50 of SCC4 cells was 33.88 μg/mL, which was also lower than the 11.53 μg/mL of SCC9 cells (Figure 8B). These *in vitro* experimental results directly support the potential value of the MCSS model in predicting the sensitivity of OSCC treatment, providing important experimental evidence for cross cancer validation results.

Furthermore, the expression level of CA9 was reduced by shRNA technology, and confirmed the knockdown efficiency by Western blot analysis. CA9 protein levels were significantly reduced in both SCC9 and SCC4 cells transfected with CA9-specific shRNA compared to control shRNA (Figures 8C,D). We then assessed the functional consequences of CA9 knockdown on OSCC cell behavior. Colony formation assays revealed that CA9 knockdown significantly restrained the clonogenic potential of both SCC9 and SCC4 cells, as evidenced by fewer colony formations (P < 0.05) (Figure 8E). The Transwell invasion experiment results also demonstrated that CA-9 knockout significantly inhibited the invasion ability of SCC9 and SCC4 cells (P < 0.05) (Figure 8F). Furthermore, wound healing assays demonstrated that CA9 knockdown substantially impaired the migratory capacity of SCC9 (Figure 8G) and SCC4 (Figure 8H) cells, with slower wound closure compared to control cells. These results collectively demonstrate the pivotal role of CA9 in promoting OSCC cell proliferation and migration. The observed inhibitory actions of CA9 knockdown on these malignant phenotypes further support our previous findings and strengthen the feasibility for targeting CA9 as a potential treating strategy in OSCC, particularly in combination with docetaxel.

## 4 Discussion

OSCC carcinogenesis and progression are propelled by the cumulation of genetic and epigenetic alterations, which diminish the survival probabilities of patients with oral malignancies (Balakittnen et al., 2023; Huang et al., 2020). Early diagnosis is pivotal for enhancing the survival rates of those afflicted with OSCC (Tan et al., 2023). Consequently, the identification of biomarkers for precancerous lesions and cancer development, coupled with the construction of predictive models for the disease, holds significant importance for improving the management of OSCC. Recently, high-throughput sequencing in conjunction with multi-omics analysis has demonstrated its potential in disease screening, diagnosis, staging, prognosis, and personalized drug therapy (Li and Wang, 2021; Zhang and Li, 2023). Moreover, the integration of multi-omics-based clustering algorithms with an understanding of pathological processes aids in the molecular typing of cancer and in enhancing the survival predictions and therapeutic outcomes for different subtypes (Collisson et al., 2019; Heo et al., 2021). For example, Yang et al. (2023) identified three HNSCC immune subtypes using immune-related gene expression and somatic mutation data, and then developed a novel, highly accurate, and interpretable machine learning-based immune subtyping predictive system. In this study, we employed an integrated multi-omics approach for an in-depth analysis of OSCC. By combining transcriptomic data-encompassing the expression levels of mRNA, lncRNA, and miRNA - as well as epigenetic characteristics (DNA methylation) and genetic mutation information, we meticulously classified OSCC patient samples using ten advanced multi-omics consensus clustering algorithms. Ultimately, three OSCC subtypes were determined, each with distinct genetic and immunological features. Subsequently, we selected the ML algorithms with the optimal predictive performance to construct a comprehensive clinical prediction model (MSCC) specific to OSCC subtypes. This model excels not only in diagnostic accuracy and prognostic reliability but also provides potential therapeutic drug options through its precise scoring system, thereby robustly supporting personalized medicine.

This research has delineated three unique subtypes of OSCC across various dimensions, showing a significant correlation with genetic profiles. The multi-omics clustering analysis has uncovered the top 10 factors associated with OS. A plethora of studies has illustrated the disrupted mRNA and miRNA expression in OSCC (Bouaoud et al., 2022; Khan et al., 2020). We detected mRNAs (LCE3A, LCE3D, LCE3E, CRCT1, among others) and miRNAs (LCE3D, SPRR2B, PRR3) associated with skin barrier functionality (Jalali et al., 2024; Niehues et al., 2023), despite no current research linking OSCC to skin functionality. Non-coding RNAs serve as critical regulators of diverse cellular processes such as proliferation, migration, invasion, apoptosis, and resistance to chemotherapy (Balakittnen et al., 2023). Notably, LINC00958 (Wang F. et al., 2020) has been recognized as an oncogenic gene with marked upregulation in OSCC tissues. Additionally, PCED1B-AS1, identified in a spectrum of cancers (Liu et al., 2022; Yao et al., 2020), is underscored in this study for its relevance to OSCC. In the context of OS-related DNA methylation anomalies in OSCC, cg10474350 has been noted in esophageal (Peng et al., 2021) and hepatic cancer (Zhang et al., 2020), indicating a potential novel methylation marker for OSCC prediction. Various mutational sites are established to influence tumor growth and invasion, including the inactivation of tumor suppressor genes TP53 (Voskarides and Giannopoulou, 2023) and CDKN2A (Gaździcka et al., 2020), which may precipitate oral carcinogenesis. The three OS subtypes also reflect the intricate interplay of transcriptional and epigenetic information. For example, FOXM1, prominently enriched in CS2, modulates several cancer cell attributes, including proliferation, metastasis, and relapse (Khan M. M. et al., 2023). A thorough examination of transcription factors and potential chromatin remodeling regulators has identified elements significantly linked to OSCC, which are already regarded as valid targets for therapeutic intervention in oncology.

Currently, OC typing is predominantly grounded in pathological histology, with OSCC representing the principal subtype (Roi et al., 2020). OSCC is further categorized into various subtypes, comprising a basal subtype akin to basal cells of the human airway epithelium (55%), a mesenchymal subtype engaged in epithelial-to-mesenchymal transition (EMT) (33%), an atypical subtype strongly linked to HPV (4%), and a classical subtype associated with smoking and xenobiotic metabolism (9%) (Chai et al., 2020). Our investigation advances the molecular stratification of OSCC, with each subtype presenting distinctive biological traits and pathway activities that exhibit varied responses to diverse treatment modalities. The OS times for subtypes CS3, CS2, and CS1 progressively diminish, while the enrichment scores for pathways such as ErbB signaling, Lysine degradation, multi-species Apoptosis, and proliferation-associated mitotic spindle and mTORC1 signaling escalate. This indicates that the activation of these pathways intensifies with disease progression. The aberrant signaling by ERBB family members, driven by genetic mutations that abnormally activate tyrosine kinase, can propel tumor initiation, progression, and severity, concurrently undermining the antitumor immune response by modulating the tumor microenvironment’s immune profile (Kumagai et al., 2021). The Lysine degradation pathway predominantly unfolds within the mitochondria, with the implicated enzymes localized in both mitochondrial and cytoplasmic compartments (Leandro and Houten, 2020), and mitochondria are notably correlated with cancer progression and drug resistance (Bai et al., 2022). However, studies elucidating these relationships have yet to emerge. Furthermore, the paradoxical presence of a pro-apoptotic state in OSCC has piqued the interest of numerous researchers, seemingly conflicting with the established characteristics of cancer (He et al., 2022). Within the CS1 subtype, we observed a significant downregulation of pathways including primary bile acid biosynthesis, cholesterol metabolism, and PPAR signaling relative to the other groups. Typically, rapidly proliferating cancer cells necessitate elevated cholesterol levels to foster membrane biogenesis and other cellular functions, and an increase in cholesterol content has been noted in numerous OSCC cases (Chan et al., 2023). The dysregulated cholesterol metabolism observed in the advanced stages of the CS1 subtype warrants further investigation.

Research indicates that primary bile acids can accumulate in T cells and induce DNA damage, with the downregulation of pathways such as primary bile acid biosynthesis in CS1 suggesting potential impairment of immune cell function (Varanasi et al., 2025). Concurrently, elevated levels of T_cells_CD4_memory_resting and activated NK cells highlight the complex immune phenotype of CS1. As a key immunosuppressive cytokine in the TIME, VEGFA orchestrates immune suppression through multiple mechanisms, such as inhibition of dendritic cell maturation, and modulation of T-cell exhaustion (Patel et al., 2023). The significant negative enrichment of VEGFA signaling in CS2 indicates heightened activation of immune-stimulatory mechanisms within this subtype’s TIME. Immune cell profiling further confirms substantial enrichment of critical effector populations in CS2, including naive B cells, CD8^+^ effector T cells, CD4^+^ memory-activated T cells, and classically pro-inflammatory M1 macrophages. Notably, CS2 exhibits marked upregulation of IC molecules such as PDCD1 (PD-1), CD274 (PD-L1), PDCD1LG2, CTLA4, TNFRSF9, and TNFRSF4. These molecules are predominantly expressed on T cells of the adaptive immune system and cells of the innate immune system (Zhang and Zheng, 2020). The indicates that the CS2 subgroup may be more receptive to immune reactions. In contrast, the cell cycle pathway, which regulates cell proliferation and division, shows negative enrichment in radiotherapy-predicted pathways in CS3, implying impaired tumor cell cycle progression and reduced proliferative capacity, potentially leading to decreased sensitivity to radiotherapy (Sun et al., 2021). Collectively, the distinct immune profiles among OSCC subtypes underscore the clinical imperative for molecular subtype-guided personalized treatment approaches. Subsequent studies on the MSCC model have shown that the expression of numerous IMs in OSCC patients are dysregulated due to silencing or mutation, leading to changes in immune cell infiltration levels and altering TIME. Particularly in high MCSS group, levels of various immune cell subsets were significantly less than in the low MCSS group, encompassing neutrophils, diverse B cells, and T cells. Targeted blockade of ICs such as PD-1, PD-L1 has emerged as an effective strategy to enhance antitumor immune responses, and the enhanced therapeutic response associated with high IC expression has been widely reported (Li et al., 2019). However, the high MCSS group consistently demonstrates inadequate immune responses to anti-PD-L1 therapy, posing new challenges for the development of our biomarkers.

The MCSS was constructed by ML algorithms with multi-omics data. Among them, the optimal combination (StepCox [both]+plsRcox) exhibited robust performance in AUC values and C-Index across, underscoring the exceptional predictive power of the MCSS model. We identified seven model genes from the integrated cohort: TPSAB1, SPINK6, MT1X, METTL7B, CPA3, CAMK2N1, and CA9. Notably, the genes CA9 and SPINK6 were found to be markedly elevated in OSCC cells. CA9, part of the carbonic anhydrase family, expresses preferentially in malignant cells under hypoxic conditions (Guan et al., 2020). The Hypoxia pathway was also detected among the top upregulated pathways in the high MCSS group. Hypoxic conditions can foster tumor growth and inhibit the antitumor immune system; reversing this hypoxia might enhance the viability and efficacy of tumor-infiltrating T cells, potentially resensitizing tumors to immunotherapy (Jayaprakash et al., 2022). The EMT pathway, significantly upregulated in the high MCSS group, is a common feature across various cancers and is key to the invasiveness and metastatic potential of OSCC cells (Wang Z. et al., 2020). Recent studies suggest that EMT may be linked to chemoresistance in OSCC(Bai et al., 2021), offering a rationale for the treatment resistance observed in the high MCSS group. Heightened E2F activity, prevalent in many cancers, leads to the dysregulation of E2F family genes in OSCC due to genetic or epigenetic alterations, a critical factor in oncogenesis (Kassab et al., 2023; Nakajima et al., 2023). Additionally, Myc targets V1, a therapeutic target in numerous cancers, correlates with the proliferation of cancer cells (Oshi et al., 2022; Zhang et al., 2022). These pathways, once upregulated, underscore the complexity of treating the high MCSS group. Our drug prediction efforts revealed the significant potential of docetaxel. As an FDA-approved taxane-based antimitotic chemotherapeutic, docetaxel can effectively induce apoptosis and modulate intracellular immune mechanisms (Gupta et al., 2023). It has been applied across a spectrum of cancers, including OSCC (Ma et al., 2022; Mohanty et al., 2022). CRISPR-Cas9 loss-of-function screening (Meyers et al., 2017) has shown that docetaxel significantly suppresses the CA9 gene in SCC9 cells. Tagawa et al. (Tagawa et al., 2023) recently discovered that docetaxel exhibits maximal cytotoxicity to CA9-22 cells when subjected to mild hyperthermia at 41°C–42°C for 45–60 min. Further investigation is warranted to fully elucidate the impact of docetaxel on CA9. This also suggests that targeting genes associated with immune suppression represents a viable therapeutic strategy for OSCC.

## 5 Conclusion

In conclusion, our comprehensive multi-omics integrative analysis has shed light on the molecular heterogeneity and complexity of OSCC. By employing a combination of transcriptomics, epigenetics, and gene mutation data, we identified three distinct OSCC subtypes (CS1, CS2, and CS3) with unique genetic and immunological features. These subtypes exhibited significant differences in pathway enrichment, immune evasion, and potential treatment responses, highlighting the importance of precise molecular subtyping in OSCC. Furthermore, we developed a robust and superior MSCC model that integrates multiple ML methods to predict patient prognosis. The MSCC model identified seven key genes (TPSAB1, SPINK6, CPA3, MT1X, METTL7B, CAMK2N1, and CA9) that play crucial roles in OSCC progression and prognosis. Stratification analysis based on MSCC scores revealed significant differences in prognosis, epigenetic alterations of immune regulatory genes, and immune cell infiltration between groups with high and low MSCC score. The insights shed light on the mechanisms underlying the prognostic differences and the potential for personalized treatment strategies. Notably, our analysis suggests that patients in the high MSCC group may not respond favorably to ICI therapy. However, we identified docetaxel and paclitaxel as potential candidate drugs for treating this population, offering new therapeutic options for OSCC patients with poor prognosis.

In summary, our study demonstrates the power of multi-omics integrative analysis in unraveling the complex mechanisms of OSCC and provides a solid foundation for the expansion of precise molecular subtyping and personalized treatment strategies. The MSCC model, along with the identified key genes and potential therapeutic targets, holds great promise for improving early diagnosis, treatment decision-making, and ultimately, patient prognosis and survival rates in OSCC. Rigorous validation through prospective clinical trials is essential to operationalize these biological insights within routine oncology practice and drive advancements in precision medicine strategies for OSCC management.

## Data Availability

The original contributions presented in the study are included in the article/Supplementary Material, further inquiries can be directed to the corresponding author.

## Supplementary material

SUPPLEMENTARY FIGURE S1 Abundance of immune infiltrating cells with significant differences.

SUPPLEMENTARY FIGURE S2 MCSS score distribution of oral squamous cell carcinoma cell lines in DepMap database.

SUPPLEMENTARY FIGURE S3 Quantitative analysis of CA9 knockdown efficiency by Western blot.

SUPPLEMENTARY TABLE S1 The relationship between MSCC and immunomodulators.

SUPPLEMENTARY TABLE S2 MCSS score distribution of oral squamous cell carcinoma cell lines in DepMap database.
