# Supplementary figures and images for: Integrative multi-omics analysis and experimental validation identify molecular subtypes, prognostic signature, and CA9 as a therapeutic target in oral squamous cell carcinoma
Source: Front Cell Dev Biol. 2025 Jul 9;13:1629683. doi: 10.3389/fcell.2025.1629683 (PMC12283686; doi:10.3389/fcell.2025.1629683)

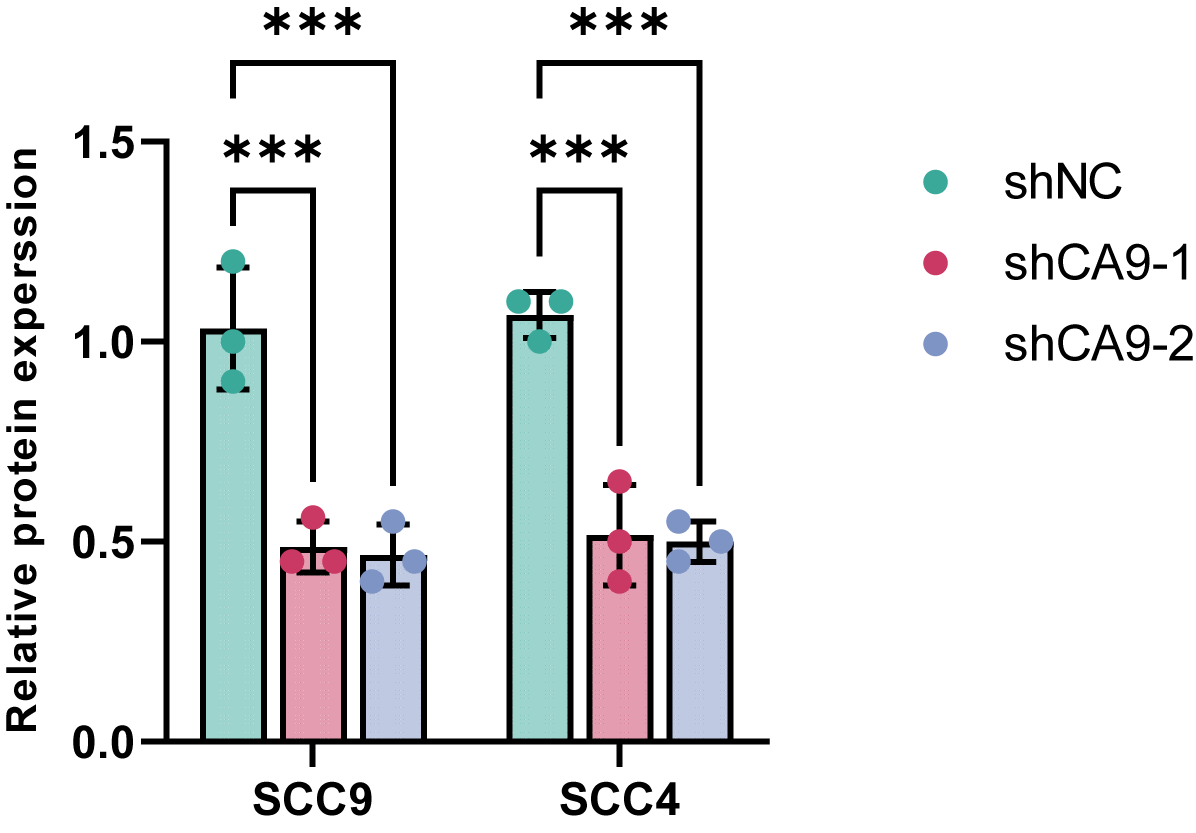

Supplement: Supplementary file 2 [file Image3.tif]

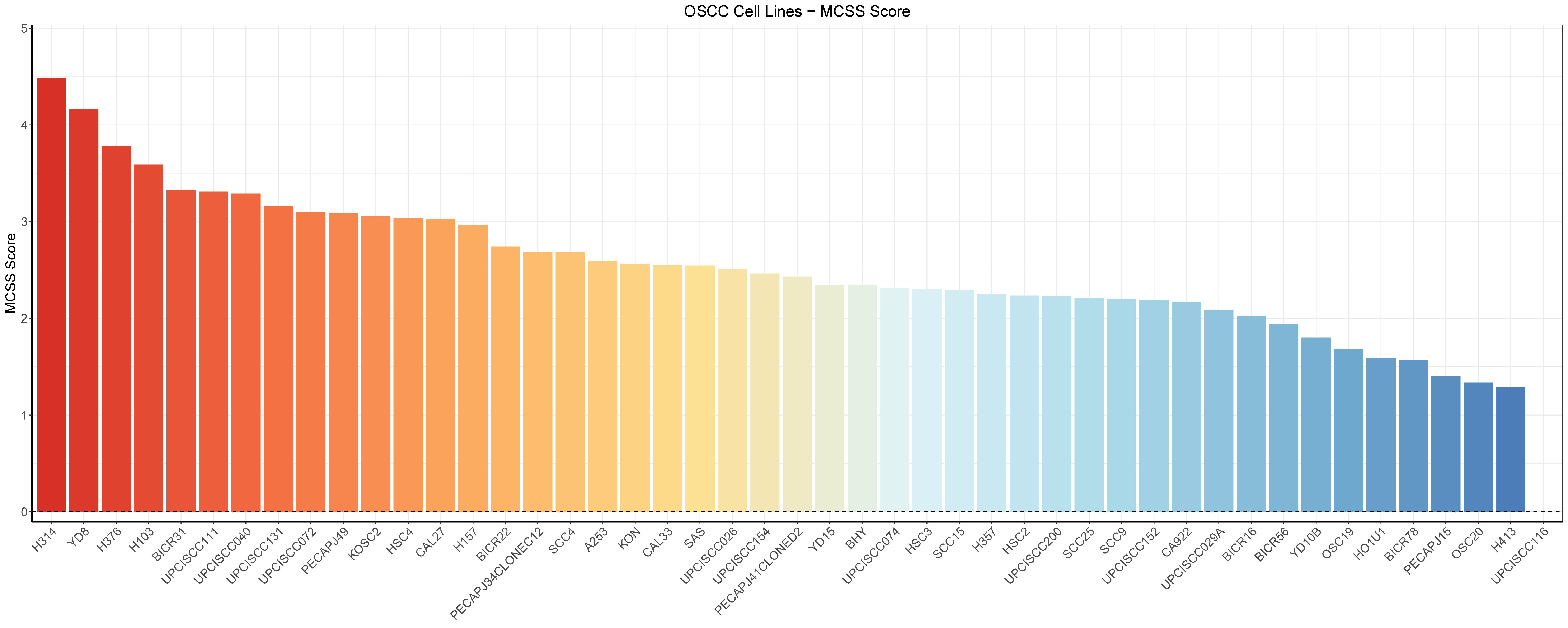

Supplement: Supplementary file 3 [file Image2.tif]

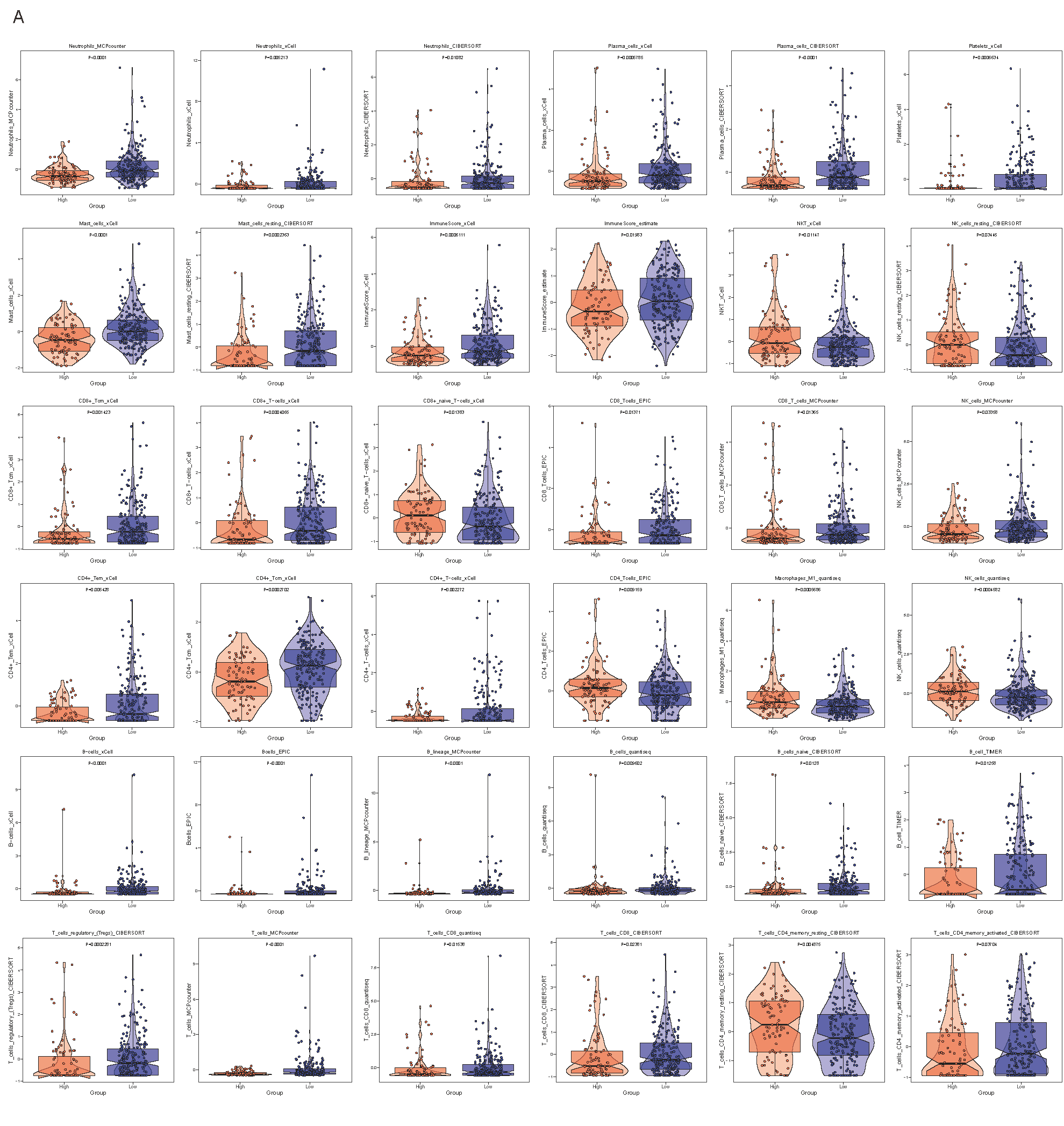

Supplement: Supplementary file 4 [file Image1.tif]
